# Alpha-1 antitrypsin promotes re-epithelialization by regulating inflammation and migration

**DOI:** 10.3389/fimmu.2025.1586039

**Published:** 2025-05-30

**Authors:** Idan Farber, Muhammad Wated, Ronen Schuster, Lihie Sheffer, Yuval Anav, Navit Ogen-Shtern, Alexandra Tsitrina, Diya Tacruri, Anna Bunin, Noah Benjamin Nagar, Maayan Hagbi Bal, Tomer Eliyahu, Dor Halpern, Boris Knyazer, Samuel Cohen, Sabri El-Saied, Eli C. Lewis, Eldad Silberstein, Erez Tsumi

**Affiliations:** ^1^ Department of Clinical Biochemistry and Pharmacology, Faculty of Health Sciences, Ben-Gurion University of the Negev, Beer-Sheva, Israel; ^2^ Department of Plastic and Reconstructive Surgery, Soroka University Medical Center, Ben-Gurion University of the Negev, Beer-Sheva, Israel; ^3^ Department of Ophthalmology, Soroka University Medical Center, Beer-Sheva, Israel; ^4^ The Skin Research Institute, The Dead-Sea and Arava Science Center, Masada, Israel; ^5^ Ben-Gurion University of the Negev, Eilat Campus, Eilat, Israel; ^6^ Microscopy and Flow Cytometry Unit, Ilse Katz Institute of Nanoscale Science, Beer-Sheva, Israel; ^7^ Department of Otolaryngology-Head and Neck Surgery, Soroka University Medical Center, Beer-Sheva, Israel

**Keywords:** adhesion molecules, corneal abrasion, desmosomes, gene expression, hemidesmosomes, inflammation, wound Healing

## Abstract

**Purpose:**

Regulation of inflammation and re-epithelialization are critical for efficient wound healing. This study explores the role of human α1-antitrypsin (hAAT), an immunomodulatory protein, in modulating inflammation and promoting re-epithelialization across various epithelial cell types.

**Methods:**

*In-vitro*, epithelial gap closure and migration assays were performed using two human epithelial cell lines—HaCaT and A549 cells with and without mitomycin C treatment. These cell lines were also used in an *in-vitro* gel-directed epithelial migration assay. Cells were treated with hAAT, and the gap area was measured using image analysis. Gene expression of inflammatory markers (IL-1β, IL-6, and TNFα) and adhesion molecules (desmoglein-1, plectin, and integrin α6β4) were analyzed using qPCR. *In-vivo*, corneal abrasions were induced in C57BL/6 mice using an Ophthalmic Burr. Mice received topical hAAT treatment immediately after injury and every 6 hours thereafter. Wound closure was assessed by applying the standard ophthalmic staining technique, fluorescein, and image analysis. Inflammatory markers and adhesion molecule expression were evaluated using qPCR and immunohistochemistry.

**Results:**

*In-vitro*, hAAT accelerated epithelial gap closure and increased migration distance, independent of cell proliferation. hAAT-treated cells also exhibited earlier peak expressions of IL-1β and IL-6. *In-vivo*, hAAT treatment accelerated corneal wound closure and resulted in a preference for IL-1Ra over IL-1β expression. hAAT also enhanced the expression of desmoglein-1, plectin, and integrin α6β4, both *in-vitro* and *in-vivo*, and increased desmoglein-1 expression in the epithelial migration zone of mouse cornea.

**Conclusions:**

hAAT enhances re-epithelialization by modulating inflammation, promoting epithelial cell migration, and regulating expression of adhesion molecules.

## Introduction

Epithelial integrity is crucial for maintaining the function of various organs and tissues. Wounds in epithelial tissues, such as skin, lungs, intestines, and cornea, might result from trauma, infection, or inflammatory conditions. These wounds trigger a complex healing process that involves two interrelated mechanisms, inflammation and re-epithelialization. During the healing process, immune cells clear debris and pathogens while simultaneously signaling epithelial cells to proliferate, migrate, and differentiate in order to restore the damaged tissue (1, 2). Inflammatory cascades and cytokines, such as IL-1β, play essential roles in promoting epithelial repair (3–6), however, uncontrolled inflammation can disrupt re-epithelialization, leading to chronic wounds, ulceration, and scarring (7, 8). In severe cases, this imbalance may cause significant tissue dysfunction and complications. Local steroid treatments are commonly used to manage excessive inflammation due to their potent anti-inflammatory effects (9), though their impact on epithelial healing varies across different tissue types.

During re-epithelialization, epithelial cells undergo significant morphological changes, as well as cytoskeletal structure and cell adhesion changes. Desmosomes and hemidesmosomes, protein complexes responsible for cell-to-cell and cell-to-extracellular matrix (ECM) adhesion, respectively, undergo dynamic modulation to facilitate epithelial cell migration. Desmosomes, composed of proteins from the desmoglein and desmocollin families, are prevalent in the corneal epithelium and play a crucial role in anchoring epithelial cells together. They provide strong adhesion and contribute to the structural integrity of migratory tissues (10). Hemidesmosomes, on the other hand, are essential for maintaining structural stability and resisting shearing forces. These structures, which contain integrin α6β4, anchor epithelial cells to the basal membrane by binding to laminin-332 and plectin (3), a protein that integrates serves as a bridge between integrins and other cytoskeletal components (10). The dynamic detachment and re-attachment of these complexes allow epithelial cells to migrate across the basal membrane, forming a leading migration zone that advances toward wound closure (11, 12).

Human α1-antitrypsin (hAAT) is a 52 kDa circulating glycoprotein from the se`rine protease inhibitor (SERPIN) protein family (13). It is the third most abundant plasma protein after immunoglobulins and albumin. hAAT levels rise physiologically during infection, inflammation and hypoxia, and are associated with accelerating inflammatory resolution, reducing bacterial burden, and advancing tissue repair (14–19). At the molecular level, hAAT directly inhibits inflammatory serine proteases, such as neutrophil elastase, thus reducing the activation of protease-activated receptors (PARs) and cleavage of pro-inflammatory cytokine precursors, like pro-IL-1β (20). Genetic hAAT deficiency (AATD) causes lung alveolar wall degradation. This clinical feature emphasizes the role that hAAT plays in epithelial integrity (21–23). Furthermore, individuals with AATD exhibit poor wound healing, which improves significantly following the initiation of replacement therapy with weekly infusions of plasma-purified hAAT (24).

Although evidence suggests that hAAT plays a role in re-epithelialization (25), its specific effects have not been thoroughly investigated, and its precise mechanism of action remains unclear. The current study investigates the effect of clinical-grade hAAT on two key processes in wound healing—inflammation, and re-epithelialization—using both *in-vitro* and *in-vivo* models.

## Materials and methods

### *In vitro* human keratinocytes gap repair assay: HaCaT cells

HaCaT cells (CLS Cell Lines Service 300493) were seeded in 12-well plates (2×10^5^) cells per well) and incubated until sub-confluence in growth medium (DMEM supplemented with 100 U/ml penicillin, 100 μg/ml streptomycin, with or without 10% FBS where indicated, all from Sartorius). A scratch was performed using a 1000-μl tip, creating a cell-free area. Wells were washed twice with PBS before adding either hAAT 0.5 mg/ml (Glassia, Kamada LTD., Nes-Ziona, Israel), epidermal growth factor (EGF) 10 ng/ml (Sigma-aldrich, Rehovot, Israel), or PBS, as a negative control for 48 hours. To improve visualization, cells were fixed in ice-cold methanol and stained with crystal violet 0.5% in 70% Methanol). Images were acquired using Zeiss Inverted microscope Axio Observer 7 and Moticam 5 camera. The gap area between cells (scratch width) was determined using ImageJ.

### *In vitro* human lung epithelial cell (A549 cells) migration assays

Scratch assay was performed using human A549 cells (ATCC #CCL-185). Cells were grown to confluence in 24-well plates, and uniform scratches were inflicted using a sterile 200-µl pipette tip, thus creating a cell-free area, as described elsewhere (25, 26). Cultures were washed twice with complete RPMI 1640 supplemented with 5% FCS (both from Biological Industries Inc., Beit Haemek, Israel). Treatments were introduced directly onto cells in 5% FCS. Cells were then treated with either PBS or hAAT 0.5 mg/ml. Mitomycin C, when used, was introduced prior to treatment (5 μM, CAS 50-07-7, Cayman Chemical, MI, USA). Images were acquired using Zeiss Inverted microscope Axio Observer 7 and Moticam 5 camera. The gap area between cells was determined using ImageJ.

Gel-directed epithelial migration assay was performed using human A549 cells (ATCC #CCL-185). A 100 mm plate was filled with agar that was prepared with complete RPMI 1640 medium, 2.5% FCS. Three uniformly distributed 1-mm punches were performed and introduced with PBS on one end, A549 lysate (×3 freeze-thaw cycles) with or without adenosine triphosphate (ATP; 50 μM, A2383-1G, Sigma-Aldrich) on the other end, and A549 cells (5×10 (4)) that were pretreated with either PBS or hAAT (0.5 mg/ml) in the center well. Cells were allowed to migrate from the center well to the well with injured cells for 24 hours, after which the gel was lifted, the cells fixed in methanol, and DAPI added to allow determination of migration distances. Images were acquired using a Zeiss Inverted microscope Axio Observer 7 and Moticam 5 camera. Migration distance was determined using ImageJ.

### Animals

Animal studies were approved by the Institutional Animal Care and Use Committee (IL-14-03-2023D) and conducted per the Guide for the Care and Use of Laboratory Animals, 8^th^ Edition. Eight-to-12–week old female C57BL/6 mice (Envigo+ Laboratories, Inc., Rehovot, Israel) were housed at a standard vivarium facility.

### *In vivo* corneal abrasion model

The model was performed as described elsewhere (27, 28). Briefly, mice were anesthetized by isoflurane inhalation (2.5% for induction, 2% for maintenance), and corneal abrasions were performed using the AlgerBrush II Ophthalmic Burr Instrument (Strong Vision Technologies, #SVT14-1-2RB) under a stereomicroscope (Stemi 305, Zeiss, Germany). The ocular burr, cleaned between applications with 70% EtOH, was applied to the center of the cornea to gently abrade the epithelium (20 mild vibrations/second, back-and-forth and sideways directions), avoiding contact with surrounding non-targeted regions of the cornea. Once a corneal wound largely bare of epithelium reached 2.5 mm in diameter, it was imaged for baseline indices by adding one drop of fluorescein onto the abraded eye (Bio Fluoro Ophthalmic Strips, BioTech Healthcare, India) and then washed with saline. The excess liquid was gently removed using a soft wipe and a ruler was placed near the eye for scaling while the eye surface was documented under blue-light through a stereo microscope (Stemi 305, Zeiss, Germany). Image analysis was conducted using ImageJ software (MedCalc Software, Ostend, Belgium). Seven µl of topical treatment was applied directly to the surface of the eye immediately after imaging and consisted of either saline, hAAT (4 mg/ml), or dexamethasone (Dexamethasone Rompharm 4 mg/ml, 4 μg/eye; West-Ward Pharmaceuticals, NJ, USA). Treatments and imaging were conducted at 0, 6, 10, and 16 hours post-injury, with follow-up continuing until full re-epithelialization was achieved.

### Histological analysis and immunohistochemistry

Animals were sacrificed at indicated time points, and eyeballs were excised and immediately immersed in 10% neutral-buffered formalin (Sigma-Aldrich). Samples were placed facing the microtome blade for paraffin embedding and then cut into 4-6 μm sections, mounted on slides, and stained with Hematoxylin and Eosin (H&E; Jackson ImmunoResearch, West Grove, PA, USA) (29).

For Immunohistochemistry, formalin-fixed, paraffin-embedded (FFPE) samples of mouse cornea were deparaffinized in 100% xylol, followed by dehydration in serial dilution of isopropanol (100%, 95%, and 70%, all from Sigma-Aldrich). After extensive washing in PBS, samples were incubated overnight with primary rabbit anti-desmoglein-1 antibody (1:200, Cat # BS-6725R, ENCO, Petach Tikvah, Israel). The next day, samples were washed 3 times with PBS for 10 min each and incubated with goat anti-rabbit Alexa Fluor 488 antibody (1:500, Cat # PA5-16891, Invitrogen) in PBS with 1 µg/ml DAPI for 1h. Finally, samples were washed with PBS 3 times, 10 min each, and mounted in Prolong Gold Antifade (P36930, Invitrogen). Imaging was performed using a Zeiss Celldiscoverer 7 (Zeiss), equipped with Plan-Apochromat ×20/0.95 objective and the appropriate filter set and light source. Image acquisition settings were similar for all samples. Images were analyzed in QuPath ver 04.4 software (30). Briefly, the corneal layer was segmented according to the DAPI threshold into 10-µm^2^ area tiles. Tiles not more than 50 µm away from the wound border were labeled as ‘migration zones’. The intensity of the desmoglein-1 channel was measured in each tile, and the resulting data was saved in CSV format.

### Gene expression analysis

Scratch assay: A549 cells were harvested for gene expression at time points 6, 12, and 24 hours from scratch. Total RNA was extracted from cultured cells using Tri-Reagent (Bio-Tri Reagent, Bio-Lab, Jerusalem, Israel) according to the manufacturer’s instructions. RNA was quantified using a NanoDrop device (Wilmington, DL, USA). Concentration-normalized RNA samples were reverse-transcribed using qScript microRNA cDNA Synthesis Kit (QuantaBio, Beverly, MA, USA). Transcript levels of various gene products were analyzed using qPCR (StepOnePlus™ Real-Time PCR System, Thermo Fisher Scientific, MA, USA). qPCR reactions were performed in triplicates for each target gene, set at a final volume of 10 μl containing 5 μl Fast SYBR™Green Master Mix (Thermo Fisher Scientific) with 12.5 ng cDNA and 0.5 μM forward and reverse primers (Biological Industries).

Mouse eye: Complete mouse eyes were collected for gene expression at time points 6, 10, and 16 hours from injury. RNA extraction was performed using Gynzol^®^ reagent (Invitrogen, Waltham, MA, USA) at 1 ml/well following manufacturer’s instructions. Subsequently, the tissue was transferred to a polytron homogenizer (gentelMACS Dissocoator, MACS Miltenyi Biotec), and the homogenized sample was loaded into a 1.5 ml RNase-free microcentrifuge tube. RNA quantification, reverse transcription, and measurement of transcript levels of various gene products were analyzed as mentioned above.

Human primer sequences (forward ‘5 to ‘3/reverse ‘5 to ‘3) for IL-1β: TCG CCA GTG AAA TGA TGG CT/GGT CGG AGA TTC GTA GCT GG, IL-6: CCA CCG GGA ACG AAA GAG AA/GAG AAG GCA ACT GGA CCG AA, Desmoglin-1: ATC CAA CCA ACT TCC GGC AT/AGT TAC GCC AGC ACC AGA AA, Plectin: CCG GGC AGT CTC TGA AGA TG/GCG TTT TCC CAA GGT TCC AG, Integrin α6β4: CTC CGC CTT CAC TTT GAG CA/TCA CCA GGT AGC CGA CGA TA and β Actin: CCT CGC CTT TGC CGA TCC/CGC GGC GAT ATC ATC ATC C for reference.

Murine primer sequences for IL-1β: CTC CAT GAG CTT TGT ACA AGG/TGC TGA TGT ACC AGT TGG GG, IL-1Ra: GAC CCT GCA AGA TGC AAG C/AGC GGA TGA AGG TAA AGC G, Desmoglein-1: GCA GTG GTG GTA ATC GTG ACC/GGA TTT TGC CTA CCG GGA GTG, Plectin: GCG GAG GAA CAG TTG CAG AA/GCC CCT TGT ACT CAT TCA GTT G, Integrin α6β4: ACT CCA TGT CTG ACG ATC TGG/GGG ACG CTG ACT TTG TCC AC and β Actin: GGT CTC AAA CAT GAT CTG GG/GGG TCA GAA GGA TTC CTA TG for reference.

Relative quantification of transcript levels was performed using the delta-delta Ct method. The variation of Ct in the reference gene across samples of a given assay was <1 cycle. The efficiency of all primer pairs was 95%-110% using a 5-point standard curve.

### Statistical analysis

All quantitative data are presented as mean ± standard error of means (SEM). The statistical significance of the differences between groups was evaluated using ANOVA followed by *post-hoc* tests for multiple comparisons. Statistical processing was performed using GraphPad Prism software (GraphPad Software, La Jolla, CA, USA). A p-value <0.05 was considered statistically significant.

## Results

### Enhanced re-epithelialization and cell migration under hAAT-rich conditions *in vitro*

**Figure 1:**
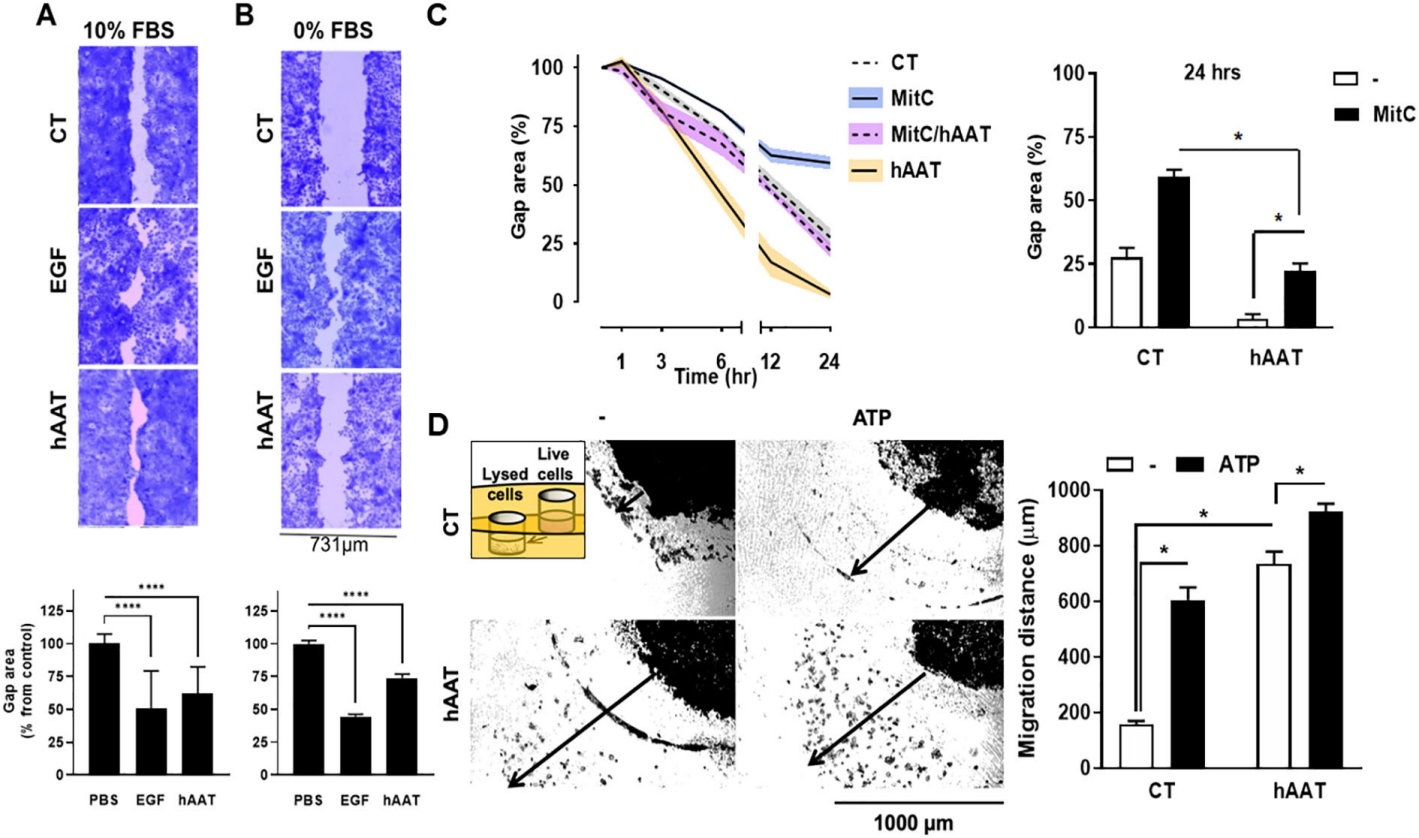
*In-vitro epithelial cell gap closure and migration* . **(A–C)** Epithelial cell gap repair assay using **(A)** HaCaT cells (human keratinocyte cell line) in 10% serum 2×105 cells/well in 12-well plates in triplicates) and **(B)** HaCaT cell in 0% serum ( *top* , representative photomicrographs; *bottom* , phosphate-buffered saline (PBS) treatment set at 100% gap area, data pooled from 2 independent experiments), and **(C)** A549 cells (human lung epithelial cell) in 5% serum (1×10^5^ cells/well in 24-well plates in triplicates) ( *left* , serial measurements, indicated time points),data pooled from 2 independent experiments. *EGF* , epidermal growth factor; *MitC* , mitomycin C. (D) Migration assay using A549 cells, set 1 mm apart from lysed A549 cells with or without added adenosine triphosphate (ATP), illustrated inset. *Left* , representative photomicrographs; *right* , percent area from initial gap area, representative data out of 3 independent experiments). *Arrow* , distance marking in image analysis. Mean±SEM. * p<0.05, **** p<0.0001.

Several models were developed to assess the effect of hAAT on epithelial gap re-epithelialization. HaCaT cells were grown to confluence in a complete 10% FBS medium (**Figure 1A**). Following the scratch wound, cells were treated with PBS, hAAT (0.5 mg/ml), or EGF (positive control). After 48 hours, hAAT-treated cells showed a significantly reduced gap area compared to PBS control (62.1 ± 4.6% vs. 100.0 ± 7.3%, mean ± SEM, respectively; P < 0.0001). To differentiate between migration and proliferation processes, the experiment was conducted under two conditions: with and without serum. Serum-free media simulates stress conditions that inhibit cellular proliferation. Under serum-free conditions, hAAT significantly accelerated gap closure compared to PBS (74.0 ± 2.8% vs. 100.0 ± 7.3% area, respectively; P < 0.0001) (**Figure 1B**). In mitomycin C (MitC)-treated A549 cells, co-incubation with hAAT resulted in significantly accelerated gap closure compared to MitC alone (22.2 ± 3.1% vs 59.3 ± 2.8% area, mean ± SEM, respectively; P < 0.05), albeit not to the extent of hAAT alone (**Figure 1C**).

In a gel migration assay, hAAT-pretreated A549 cells exhibited substantial migration towards lysed cells, irrespective of added ATP, compared to PBS control (5-fold and 6-fold increased migration for hAAT alone and hAAT/ATP, respectively; P < 0.05) (**Figure 1D**).

**Figure 2:**
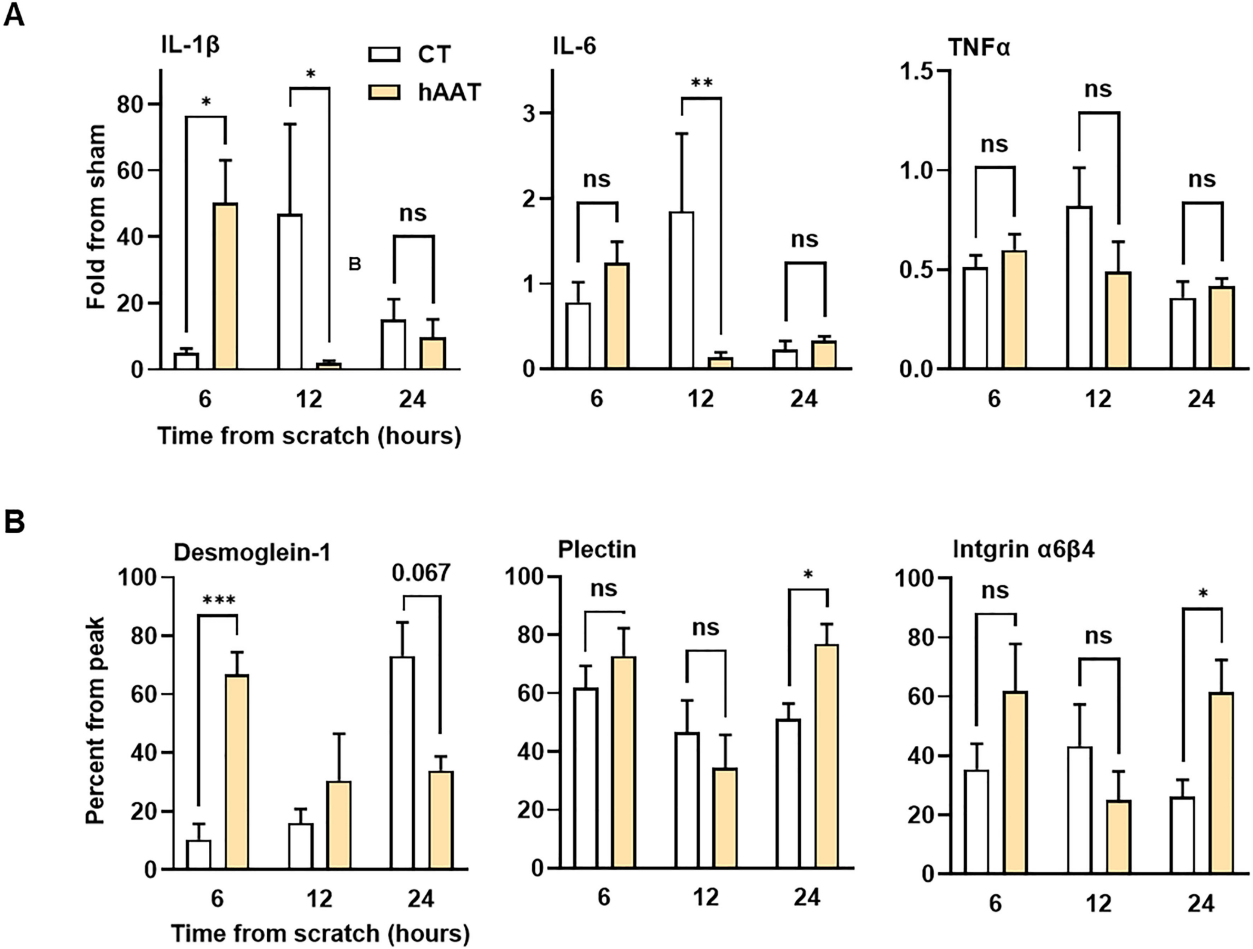
*Gene expression profile in epithelial gap closure assay.* A549 cells treated with phosphate-buffered saline (PBS) or hAAT 0.5 mg/ml in triplicates, gap inflicted at 0 hr. Cells were harvested for gene expression analysis at indicated time points. Data presented as **(A)** fold from sham and **(B)** percent from peak expression. Representative data out of 2 independent experiments. Mean± SEM; ns, not significant, *p<0.05, **p<0.01 and ****p<0.001.

Gene expression analysis of A549 epithelial cells subjected to a scratch assay and treated with either PBS or hAAT (0.5 mg/ml) revealed marked differences between groups. In the control group, IL-1β and IL-6 gene expression peaked at 12 hours, while in the hAAT-treated group, peak expression occurred earlier at 6 hours (**Figure 2A**). TNFα expression remained relatively unchanged. Desmoglein-1 expression showed a progressive increase over 24 hours in the control group, while in the hAAT group, expression peaked at 6 hours. At 24 hours, hAAT treatment significantly upregulated plectin and integrin α6β4 expression compared to the control group(**Figure 2B**).

### Topical hAAT treatment accelerates corneal wound healing and re-epithelialization *in vivo*

**Figure 3:**
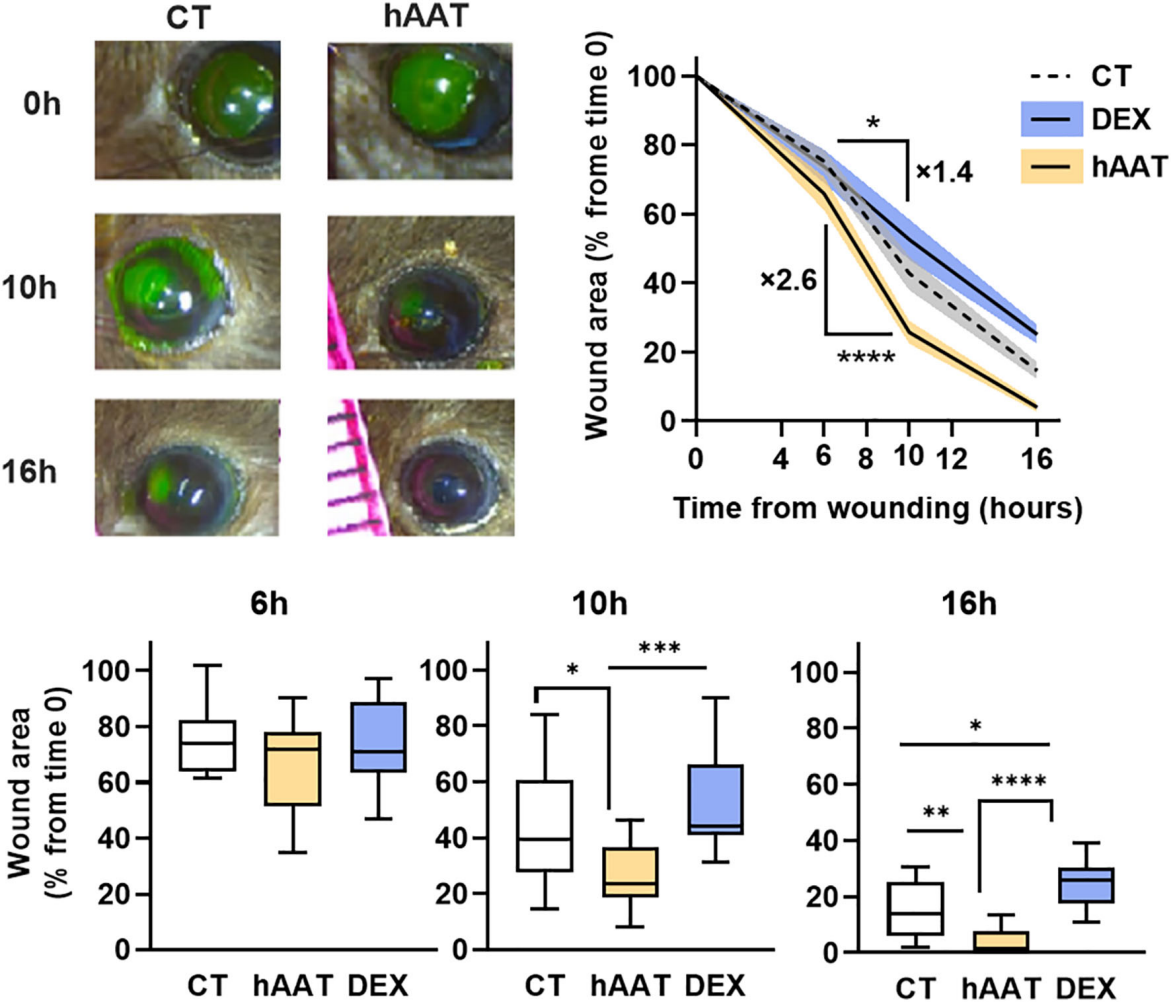
*In-vivo wound area in mouse corneal abrasion model.* Corneal abrasion performed at time 0, topical treatments provided at the time of wounding and then every 6 hours. CT, control (saline 7 µl, n=15); hAAT (4 μg/eye, n=12); DEX (dexamethasone, 4 μg/eye, n=10). Wound area was quantified at 6,10 and 16 hours, represented as percent of initial wound area. Data pooled from 4 independent experiments. Mean ± SEM, deviation represented by solid fill. Box and whiskers represent mean (min to max); * p<0.05, **p<0.01, ***p<0.001, ****p< 0.0001.

Given that hAAT enhanced gap closure in the scratch assay of epithelial cell cultures, it was important to determine whether topical hAAT would elicit a similar effect *in-vivo*. In a mouse corneal abrasion model, hAAT-treated wounds showed significantly reduced wound area at 10 and 16 hours post-injury compared to saline control (25.79 ± 11.49% vs. 42.83 ± 19.56% at 10h; 4.04 ± 4.90% vs. 14.67 ± 9.94% at 16h, respectively; mean ± SEM, P < 0.05) (**Figure 3**). To compare AAT function to the common treatment (steroids) an additional group of dexamethasone (a steroid medication) was included. Dexamethasone (DEX) treatment slowed wound closure compared to control. The hAAT group demonstrated a significantly steeper wound area reduction between 6–10 hours compared to the control (2.6-fold; P < 0.0001).

**Figure 4:**
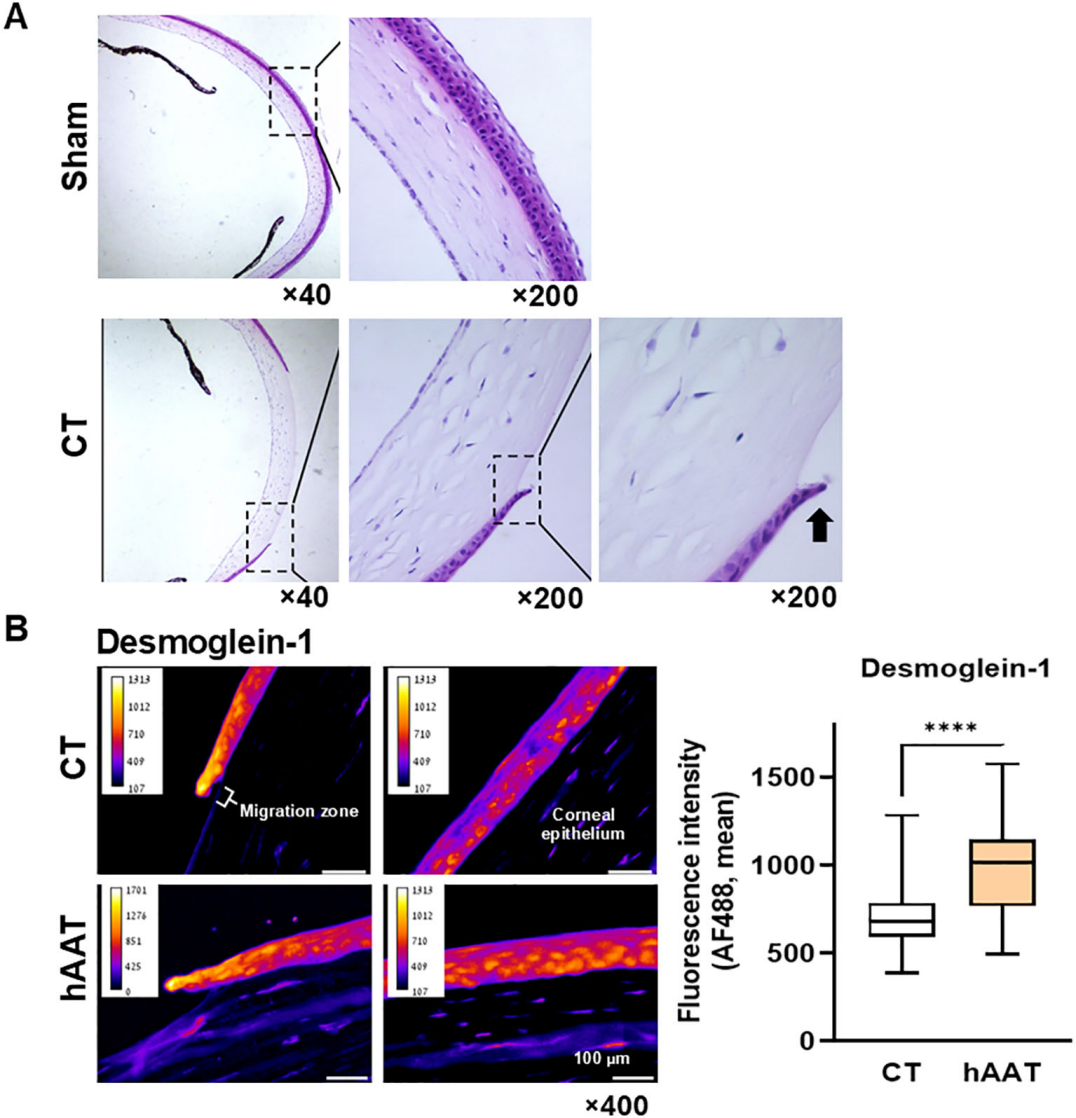
*In-vivo re-epithelialization: corneal abrasion model.* **(A)** Untreated abrasion, Hematoxylin and Eosin (H&E), 10 hrs. Representative microscopic images out of 8 samples. Radial plane. *Black arrow*, epithelial leading cell. **(B)** Desmoglein-1 immunofluorescent staining. Representative images. *Migration zone*, 50 µm from wound edge; *corneal epithelium*, adjacent corneal layer. *Right*, mean fluorescent intensity per 10 µm^2^ tiles, pooled data from 3 slides per group. Mean, ****p<0.0001.

Histological analysis revealed a selective impact on the epithelial layer while leaving the underlying stromal layer uninvolved (**Figure 4A**). Immunohistochemistry showed significantly higher desmoglein-1 levels in the migratory zone of hAAT-treated wounds compared to the control group (**Figure 4B**).

**Figure 5:**
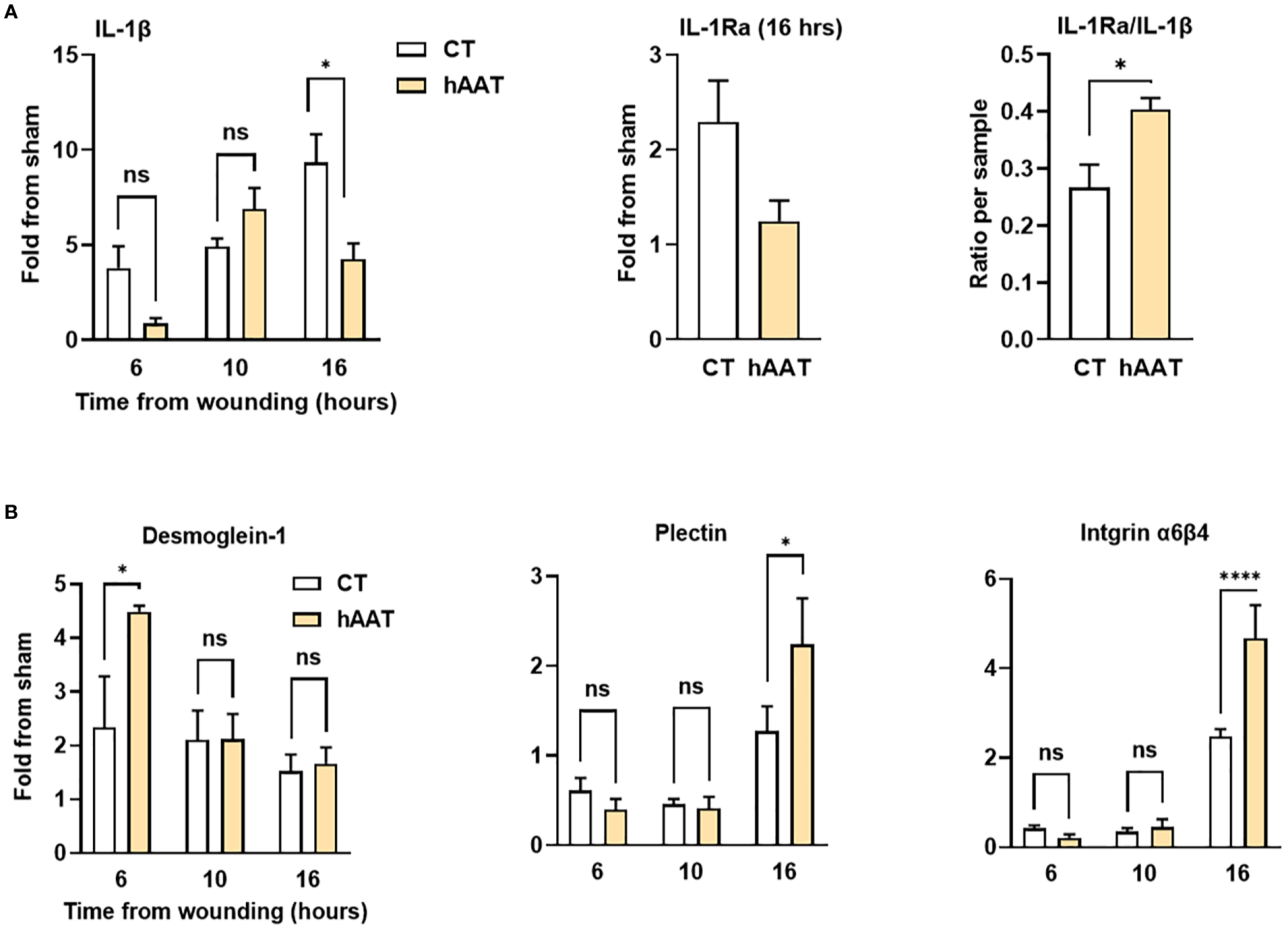
*Gene expression profile in mouse corneal abrasion model.* Corneal abrasion was performed at time 0, and topical treatments were applied at the time of injury and at 6, 10 and 16 hours post-injury. Complete eyes were collected at indicated time points for gene expression analysis. **(A)** Expression of inflammation-related genes; interleukin-1 beta (IL-1β), interleukin-1 receptor antagonist (IL-1Ra), and IL-1Ra/IL-1β ratio. **(B)** Expression of genes related to adhesion molecules; Desmoglein-1, Plectin, and Integrin α6β4. Data are presented as fold change from sham or as ratio per sample. Representative results from three independent experiments. Mean ± SEM; ns, not significant; *p < 0.05, ****p < 0.0001.

To further examine the underlying mechanisms that allow rapid epithelization under topical hAAT treatment, gene expression analysis was performed (**Figure 5**; *sham*, normal eye). The control group showed steadily increasing IL-1β expression, while the hAAT group peaked at 10 hours and decreased thereafter (**Figure 5A**). At 16 hours, hAAT treatment induced a predominance of IL-1Ra over IL-1β (P = 0.042).

In the control group, desmoglein-1 expression increased 2-fold within 6 hours post-injury and remained steady thereafter (**Figure 5B**). hAAT treatment induced a significant 4.5-fold surge in desmoglein-1 expression at 6 hours (P = 0.029). Plectin and integrin α6β4 expression increased at 16 hours in both groups, with significantly higher levels in hAAT-treated wounds (2.2 ± 0.5-fold vs. 1.5 ± 0.2-fold for plectin; 4.8 ± 1.3-fold vs. 2.4 ± 0.2-fold for integrin α6β4; hAAT-treated and control respectively, P < 0.05). This finding matches the scratch assay gene expression results.

## Discussion

The present study investigated the effect of clinical-grade hAAT on two major processes in wound healing: inflammation and re-epithelialization. Using both *in-vitro* human epithelial cell lines (HaCaT and A549) and an *in-vivo* mouse corneal abrasion model, we demonstrate that hAAT accelerates re-epithelialization primarily through enhancing epithelial cell migration rather than proliferation. Specifically, hAAT appears to promote the migration of epithelial cells toward sites of injury and cell death. These findings were supported by the observation of a clearly defined migration zone in the *in-vivo* model, characterized by well-adhered epithelial cells resembling those of a healthy corneal epithelium. Moreover, hAAT administration improved the quality of wound closure at an early stage in both *in-vivo* and *in-vitro* models.

hAAT has been found to shift the inflammatory process in the cornea, resulting in an earlier, more efficient, and shorter duration of inflammation and therefore faster resolution of inflammation. Gene expression analysis in the epithelial scratch assay and the corneal abrasion model revealed that the inflammatory phase occurred earlier in hAAT-treated cells and mice than in controls, shown by an earlier peak in IL-1β and IL-6 expression in hAAT-treated cells. The faster resolution of inflammation in hAAT-treated mice, evidenced by the preferential expression of IL-1Ra relative to IL-1β, may create a more favorable environment for epithelial cell migration (14, 15, 20).

Schuster et al. reported that hAAT enhances the expression and production of IL-1Ra in LPS-stimulated murine peritoneal macrophages, requiring nuclear translocation of the NF-κB family member, p65 (26). These findings suggest that hAAT does not block inflammation, but rather it redirects the inflammatory surge toward a resolution profile characterized by the induction of anti-inflammatory gene expression. Consistent with this notion, we found that hAAT allowed a modest increase in the pro-inflammatory markers IL-1β, IL-6, and TNFα rather than completely blocking them, suggesting a regulated initiation of the inflammatory phase. Significantly, at later stages, while control mice continued to exhibit a sustained inflammatory profile dominated by IL-1β and IL-6, hAAT-treated mice exhibited significantly reduced levels of these cytokines. Notably, hAAT treatment also led to a gradual predominance of IL-1Ra, a pro-resolution cytokine that negates IL-1β activities. The comparison between hAAT and dexamethasone (DEX) treatments further underscores this phenomenon. DEX, a potent anti-inflammatory corticosteroid that blocks p65 nuclear translocation and is frequently used in corneal abrasion treatment (9), significantly delayed corneal abrasion healing in our study. The current clinical treatment with steroids seeks to reduce inflammation, lowering the risk of complications caused by inflammatory injury, at the expense of delayed epithelialization (31). This study suggests that local therapy with hAAT may allow for both early inflammation resolution and accelerated wound healing.

Interestingly, the research findings suggest that hAAT not only accelerates the migration of epithelial cells, leading to faster healing of the cornea, but also enhances the quality of the healing process in its early phases. Desmoglein-1, a key component of desmosomes, was significantly upregulated in hAAT-treated mice compared to controls at an earlier stage of wound healing. Immunohistochemical staining revealed elevated desmoglein-1 protein levels specifically within the migration zone of hAAT-treated corneal epithelium. This localized increase, relative to other regions of corneal epithelial, supports the notion that hAAT modulates the inflammatory response in a manner that promotes desmosome formation and facilitates epithelial migration. The short early inflammatory process in the presence of hAAT may promote desmoglein-1 production, facilitating cell-to-cell connection during epithelial migration and preventing epithelial detachment, a crucial factor in the overly inflamed cornea (32). Additionally, the expression of plectin and integrin α6β4, components of the hemidesmosomes that anchor the epithelium to the basal membrane, increased in the final phase of migration of hAAT-treated cells and mice. This suggests that hAAT may also facilitate hemidesmosome formation thereby enhancing epithelial stability and promoting wound closure. The wound-stabilizing properties of hAAT observed in our study are consistent with findings in other tissue types. Notably, Gimmon et al. demonstrated accelerated skin wound healing with systemic hAAT treatment, showcasing enhanced wound stability even during early suture removal when compared to controls (25). Our results extend these observations to the corneal context, suggesting that the ability of hAAT to promote wound stability in early healing stages may be a generalizable feature across different epithelial tissues.

The study invites a deeper exploration of the potential mechanisms by which hAAT may accelerate cell migration of some cell types but inhibit cell migration of other cell types. For instance, hAAT expedites immune dendritic cell migration, likely by promoting the expression of membrane CCR7, the homing receptor that senses draining lymph node–derived chemokines (16, 17). Similarly, hAAT accelerates the revascularization process in the context of endothelial cell survival, migration, and spatial organization (33). In contrast, hAAT downregulates MCP-1 expression, the chemokine responsible for the migration of monocytes and macrophages (15), and neutrophil migration, both by directly binding to their major chemokine, IL-8 (34), and by inhibiting serine proteases employed by neutrophils for extravasation and tissue degradation along their migratory path (35). Nonetheless, neutrophils can neutralize hAAT by exerting a local oxidative burst and are known to store hAAT in their granules for release upon activation (36). In this context, the observation that hAAT decreases bacterial burden (19) aligns with its reported ability to inhibit neutrophil apoptosis (37). In the setting of wound repair, the expression of chemokine receptors is as crucial as the chemokines themselves (38). Here, lysed cells were used to guide the migration of live epithelial cells, finding that cells migrated faster toward the injured focus point; since the dead epithelial cells are no longer influenced by exogenous treatments, it may be suggested that the effect of hAAT was directed at chemokine receptors on the migrating epithelial cells. Further studies should explore the potential impact of hAAT on growth factors involved in wound healing, such as EGF and TGFβ (39). Notably, Nerve Growth Factor (NGF) is recognized as a key player in corneal ulcer healing due to its role in epithelial regeneration and neural repair. Although no direct interaction between hAAT and NGF has been reported to date, this potential relationship warrants further investigation.

Importantly, hAAT enhances epithelial cell migration independent of cell proliferation, suggesting that it may be considered a therapeutic agent for promoting wound healing in conditions where cell division is significantly compromised, such as in diabetic wounds or cases of chemotherapy-treated patients (40, 41). It is possible that hAAT modifies the ECM in a manner that promotes epithelial cell migration, as in the case of the perseverance of fibronectin under hAAT-rich conditions (42).

In considering cancerous epithelial migration, study results are under debate. In some instances, hAAT was shown to facilitate lung adenocarcinoma metastasis (42–45), while others provide evidence that lung and colon metastasis are dramatically blocked by hAAT in the whole animal (46–48), seemingly by activation of anti-tumor NK cell activities (49). Brami et al. directly explored the ability of hAAT to accelerate re-epithelialization in human colonic epithelial cells exposed to immunosuppressive drugs and to sera from patients that have undergone bone-marrow transplantation under significant immunosuppression (50, 51). These findings raise the possibility that patients undergoing chemotherapy could benefit from hAAT treatment to support the restoration of epithelial barrier integrity—an effect that may also hold relevance for other protein-losing enteropathies.

In conclusion, unlike conventional steroidal treatments, hAAT promotes re-epithelialization by fine-tuning the inflammatory response—inducing an earlier onset of resolution and enabling early and efficient epithelial cell migration, independent of cell proliferation capacity. These findings underscore the multifaceted role of hAAT in orchestrating the inflammatory response, enhancing desmosome formation and hemidesmosome assembly, and ultimately accelerating epithelial migration and wound healing.

## Data Availability

The raw data supporting the conclusions of this article will be made available by the authors, without undue reservation.
